# Invasive freshwater snails form novel microbial relationships

**DOI:** 10.1111/eva.13158

**Published:** 2020-11-20

**Authors:** Laura Bankers, Dylan Dahan, Maurine Neiman, Claire Adrian‐Tucci, Crystal Frost, Gregory D. D. Hurst, Kayla C. King

**Affiliations:** ^1^ Department of Biology University of Iowa Iowa City IA USA; ^2^ University of Colorado ‐ Anschutz Medical Campus Aurora CO USA; ^3^ School of Medicine Stanford University Stanford CA USA; ^4^ Institute of Integrative Biology University of Liverpool Liverpool UK; ^5^ Department of Zoology University of Oxford Oxford UK

**Keywords:** coevolution, infection, invasion, microbiome, *Potamopyrgus antipodarum*, symbiosis

## Abstract

Resident microbes (microbiota) can shape host organismal function and adaptation in the face of environmental change. Invasion of new habitats exposes hosts to novel selection pressures, but little is known about the impact on microbiota and the host–microbiome relationship (e.g., how rapidly new microbial associations are formed, whether microbes influence invasion success). We used high‐throughput 16S rRNA sequencing of New Zealand (native) and European (invasive) populations of the freshwater snail *Potamopyrgus antipodarum* and found that while invaders do carry over some core microbial taxa from New Zealand, their microbial community is largely distinct. This finding highlights that invasions can result in the formation of novel host–microbiome relationships. We further show that the native microbiome is composed of fewer core microbes than the microbiome of invasive snails, suggesting that the microbiota is streamlined to a narrower set of core members. Furthermore, native snails exhibit relatively low alpha diversity but high inter‐individual variation, whereas invasive snails have higher alpha diversity but are relatively similar to each other. Together, our findings demonstrate that microbiota comparisons across native and invasive populations can reveal the impact of a long coevolutionary history and specialization of microbes in the native host range, as well as new associations occurring after invasion. We lay essential groundwork for understanding how microbial relationships affect invasion success and how microbes may be utilized in the control of invasive hosts.

## 1 INTRODUCTION

All organisms are home to numerous microbes. The relationship between hosts and their microbial communities (microbiota) can play a role in key host functions (Archie & Theis, 2011; Brucker & Bordenstein, 2012; Dai et al., 2016) from nutrition (Adams and Douglas, 1997) to behaviour (Sharon et al., 2010) and immune responses (Koch & Schmid‐Hempel, 2011). The microbiota has also been shown to impact host evolution (Aires et al., 2016; Brucker & Bordenstein, 2013). Microbial community composition can be determined by host traits such as genotype (Bowen et al., 2017; Parker et al., 2017), sex (Cong et al., 2016), infection of the host by parasites or pathogens (Jani & Briggs, 2014), environmental conditions (Aires et al., 2016; Ramirez et al., 2019; Wong & Rawls, 2012) and genotype by environment interactions (Macke et al., 2017). Therefore, both host organisms and the microbes on which they depend for vital processes can be impacted by environmental changes, with potential consequences for host adaptation (Corbin et al., 2017).

Host‐associated microbes are expected to facilitate rapid adaptation, but the extent to which they may help invaders adapt to new habitats is relatively understudied (except see: (Aires et al., 2016; Coats & Rumpho, 2014; Kelly et al., 2014; Zilber‐Rosenberg & Rosenberg, 2008)). When species invade novel habitats, they are exposed to new environmental conditions and selection pressures. Because host and microbe fitness can be mutually dependent, invasion is predicted to influence both the host and its microbiome (Aires et al., 2016). The fitness effects of microbes on their host may affect invasion success and the potential for subsequent adaptation to a new habitat (Coats & Rumpho, 2014). The performance of introduced species could also be altered by the availability of microbes outside their native range if there are core microbes the host needs to thrive. If host‐associated microbes are not all carried over from the native environment, are lost upon invasion, and/or unavailable in the new environment, the microbiome diversity of invasive lineages could be lower than their native counterparts. The invader's microbiome might thus only represent a subset of microbial taxa residing in the native host lineages (Minard et al., 2015; Zepeda‐Paulo et al., 2018). This pattern is directly analogous to the loss of genetic diversity that often characterizes invasive lineages (Minard et al., 2015; Städler et al., 2005). Whether such subsampling occurs at the microbiome level remains an open question with implications for host fitness in the new environment.

Invasion may affect the level of microbial community diversity in invasive hosts relative to hosts in the native range. Lower alpha diversity in invasive populations could result from the comparatively short time period for coevolution between hosts and newly acquired microbes (Minard et al., 2015; Zepeda‐Paulo et al., 2018). If suitable beneficial microbes are not now available, the effects of colonization on microbial community composition could be harmful for the host. Alternatively, diversity may be higher within invasive hosts as they form new relationships with microbes (Himler et al., 2011) or if there is decreased immune‐mediated control of novel microbes (Foster et al., 2017). By this logic, we might therefore expect native host individuals to have a less rich microbiota because established and stable microbial communities, in which novel competitive interactions between microbes have long since played out, are likely to be of lower diversity (Ghoul & Mitri, 2016). This prediction might be particularly likely to hold whether the host has evolved to cultivate beneficial associations (Foster et al., 2017), addressing whether these hypotheses require characterization of how invasion impacts microbial diversity and composition within and among populations.

Molluscs are one of the most species‐rich phyla (Smith et al., 2011). They represent an ecologically and economically important class of invaders (Alonso & Castro‐Díez, 2008; Clusa et al., 2017); however, little is known about the relationships between molluscs and their resident microbes with the exception of a handful of recent studies (King et al., 2012; Neu et al., 2019). Here, we leveraged a powerful model system for host‐parasite coevolution, the New Zealand freshwater snail *Potamopyrgus antipodarum*, to characterize the impact of invasion on host microbiota. There is substantial across‐lake population variation in microbiome composition among native *P. antipodarum* (Takacs‐Vesbach et al., 2016). *Potamopyrgus antipodarum* in native populations also feature wide individual‐level variation in reproductive mode (sexual versus asexual) and ploidy level (sexual snails are diploid while asexual snails are polyploid (triploid or tetraploid) (Lively, 1987; Neiman et al., 2011)). They have also been demonstrated to be coevolving with sterilizing trematode parasites (Dybdahl & Lively, 1996; King et al., 2009). By contrast, invasive lineages of *P*. *antipodarum* in Europe and North America are polyploid asexuals with reduced genetic variation compared with the native range (Städler et al., 2005; Verhaegen et al., 2018) and have escaped from parasite infection (Alonso & Castro‐Díez, 2012; Żbikowski & Żbikowska, 2009).

We conducted 16S rRNA sequencing of field‐collected *P. antipodarum* across New Zealand and Europe to assess changes in microbiota diversity and composition across and within native and invasive host populations. We hypothesized several nonmutually exclusive possible outcomes: (a) microbial community composition (alpha diversity) of invasive *P. antipodarum* would contain a subset of the taxa observed in native snails (analogous to patterns often described for genetic variation postinvasion). Lower alpha diversity could consequently rise because invasive snails have less time to form new microbial relationships or some native microbial taxa are not now available to acquire; (b) microbe taxa would be at similar relative abundance in invasive populations if snails actively promote taxa in their native environment and these are available to acquire in the invasive range; and (c) invaders also could have fewer conserved microbes than native counterparts due to relatively recent contact in the new environment and less time for specialization. We also address associations between the fundamental and often‐variable organismal traits of sex, reproductive mode and infection status on microbiota within native host populations. More broadly, this study provides a starting point to evaluate whether and how microbes could be utilized to understand the biology of invasive host species and potentially how to control them.

### 1.1 Study System

Native New Zealand *P. antipodarum* are primarily found in freshwater lakes and streams (Winterbourn, 1968). These snails have gained prominence as a model for studying the evolutionary maintenance of sexual reproduction (Lively, 1987; Neiman et al., 2011), as well as host‐parasite coevolution (King et al., 2009; Lively, 1987) with a sterilizing trematode parasite, *Atriophallophorus winterbourni* (formerly *Microphallus livelyi*) (Blasco‐Costa et al., 2020; King et al., 2009; Lively, 1987). Multiple triploid and tetraploid asexual lineages have been separately derived from diploid sexual conspecifics (Lively, 1987; Neiman et al., 2011). A previous study suggested that the microbiome composition of these snails varies substantially among native populations and between sexual and asexual forms (Takacs‐Vesbach et al., 2016). However, these snails were either laboratory‐cultured or housed in a laboratory for several months before harvest (Maurine Neiman, personal communication), and whether the results in (Takacs‐Vesbach et al., 2016) hold in snails sampled directly from the field remains unclear.

Asexual lineages of *P. antipodarum* are successful invaders. These snails can survive a range of harsh conditions that may facilitate travel to and establish in new habitats (Alonso & Castro‐Díez, 2008; Gérard et al., 2003). Invaders are susceptible to few biological enemies (Alonso & Castro‐Díez, 2008), passing alive through the digestive systems of trout (Vinson & Baker, 2008) and are rarely infected by parasites (Gérard et al., 2003). Once they invade, these snails can influence ecosystems due to their rapid population growth (Alonso & Castro‐Díez, 2008; Quinn et al., 1996), high population densities (Alonso & Castro‐Díez, 2012; Hall et al., 2003) and competitive exclusion of native invertebrates (Alonso & Castro‐Díez, 2008; Quinn et al., 1996). Invasive *P. antipodarum* can even harm the native predators that ingest them (Vinson & Baker, 2008).

## 2 MATERIALS AND METHODS

### 2.1 Sample collection, processing and sequencing

We collected adult *Potamopyrgus antipodarum* during warm seasons from shallow (lake depth <1 m) rocks and vegetation from three New Zealand collection sites (early January 2015) and ten European collection sites (late May 2016) (Table 1; Figure S1). Snails were maintained in identical 15‐L tanks separated by source population in a 16°C room with a 16:8 hr light:dark cycle for less than one month before dissection. Snails were fed dried *Spirulina* cyanobacteria ad libitum (Zachar & Neiman, 2013).

**Table 1:** Sample collection sites

Country	Location	Geographic Coordinates	Reproductive Mode	Infection	Sex	No. Snails
New Zealand	Lake Alexandrina	43°56'12.9"S, 170°27'35.4"E	Sexual	Infected	Female	9
			Sexual	Infected	Male	2
			Sexual	Uninfected	Female	22
			Sexual	Uninfected	Male	19
			Asexual	Unknown	Female	16
			Asexual	Infected	Female	10
			Asexual	Uninfected	Female	20
New Zealand	Lake Kaniere	42°48'21.8"S, 171°7'40.5"E	Sexual	Infected	Female	2
			Sexual	Uninfected	Female	6
New Zealand	Lake Rotoroa	41°47'44.5"S, 172°36'22.4"E	Sexual	Infected	Female	1
			Sexual	Uninfected	Female	10
			Asexual	Infected	Female	1
			Asexual	Uninfected	Female	10
Austria	Lake Mondsee	47°50'37.7"N, 13°20'30.7"E	Asexual	Uninfected	Female	17
Belgium	Brakel	50°48'12.1"N, 3°45'20.9"E	Asexual	Uninfected	Female	12
Belgium	Geraardsbergen	50°47'30.4"N, 3°54'15.0"E	Asexual	Uninfected	Female	13
Belgium	Melle	51°1'11.6"N, 3°48'59.6"E	Asexual	Uninfected	Female	12
Germany	Wrohe	54°16'7.4"N, 9°57'35.9"E	Asexual	Uninfected	Female	3
Germany	Pulsen	54°19'17.3"N, 10°27'9.8"E	Asexual	Uninfected	Female	12
Switzerland	Lake Geneva	46°30'45.2"N, 6°36'27.4"E	Asexual	Uninfected	Female	15
United Kingdom	West Holme	50°40'20.1"N, 2°10'29.03"W	Asexual	Uninfected	Female	11
United Kingdom	Bovington	50°41'26.1"N, 2°13'29.02"W	Asexual	Uninfected	Female	12
United Kingdom	Oxford	51°45'6.5"N, 1°13'0.35"W	Asexual	Uninfected	Female	12

Note

The coordinates and location names of sample sites for both native and invasive snails are list below. Ploidy is a proxy for reproductive mode; diploids are sexual, polyploids are asexual. We also include the sex, infection status and number of individuals sequenced from each condition and location and condition.

All snails were sexed and shells were removed prior to DNA extraction and assessed for *A. winterbourni* infection based on the presence of metacercariae cysts via dissection (Bankers et al., 2017) (Table 1). Snails containing non‐*A. winterbourni* infections were excluded from the study. All metacercariae were removed from infected snails using a micropipette. After dissection, we used flow cytometry (Krist et al., 2017; Neiman et al., 2011) to determine ploidy status as a proxy for reproductive mode of New Zealand samples (diploids are sexual and polyploids are asexual). Because it is already established that the European invasive lineages are virtually all polyploid asexuals (Liu et al., 2012; Städler et al., 2005), we did not perform flow cytometry on these samples.

We used the Qiagen DNeasy Plant Mini Kit (QIAGEN Inc.) to extract DNA from whole snails (shells and metacercariae removed), following manufacturer protocol, but eluting DNA in 40 μl 100:1 TE buffer. The Plant kit was used as it better handles the polysaccharides present in snail mucus, compared with other DNA extraction kits. Samples with a 260/280 ratio >1.6 and containing >20 ng of total DNA, based on Nanodrop® 1,000 (Thermo Fisher Scientific), were analysed on a 1% agarose gel. Samples that reached our quality criteria and produced clear gel bands were shipped to the W.M. Keck Center for Comparative Functional Genomics (University of Illinois at Urbana‐Champaign) for sequencing. See Electronic Supplementary Methods in Appendix S1 for more details about sample collection and processing.

The 16S rRNA V4 region was amplified from the *P. antipodarum* microbiome gDNA using the 515F Golay‐barcoded primers and 806R primers (Apprill et al., 2015; Caporaso et al., 2011). Samples were prepared in accordance with the standard Earth Microbiome Project 16S rRNA protocol (Caporaso et al., 2012). Please see our Electronic Supplementary Methods in Appendix S1 for PCR mixtures and thermocycler conditions. gDNA was quantified using the Qubit 2.0 fluorometer (Thermo Fisher Scientific), amplicons were pooled at equimolar ratios (~240 ng per sample) and amplicon pools were cleaned using the Qiagen PCR Purification Kit (QIAGEN Inc.). The multiplexed library was quality checked and sequenced with the MiSeq 2x250 bp PE v2 protocol at the W.M. Keck Center for Comparative Functional Genomics (University of Illinois).

### 2.2 Computational and statistical analyses

We removed PhiX sequences from sequencing libraries using Bowtie2 (Langmead & Salzberg, 2012) (PhiX genome obtained from: support.illumina.com/sequencing/sequencing_software/igenome.html). Demultiplexed paired‐end fastq files were processed using DADA2 in R (3.4.0), with the suggested filtering and trimming parameters, as previously described (Callahan et al., 2016). We then merged paired‐end reads and constructed an amplicon sequence variant (ASV) table. We used the native implementation of the DADA2 Ribosomal Database Project (RDP) naïve Bayesian classifier (Cole et al., 2014) trained against the GreenGenes 13.8 release reference fasta (https://zenodo.org/record/158955#.WQsM81Pyu2w) to classify ASVs taxonomically.

We used phyloseq v. 1.16.2 to perform estimate_richness and vegan's pd function to calculate alpha diversity measurements of observed ASVs, Shannon's index, PD Whole Tree, Pielou's evenness and Chao1 (McMurdie & Holmes, 2013), and to perform ordinations using PCoA on unweighted and weighted UniFrac distance scores (Lozupone & Knight, 2005). We used the Songbird reference frame approach to calculate taxa differentials (Morton et al., 2019). Data visualization and statistical analyses were performed in R; see Electronic Supplemental Methods for details.

We controlled for effects of snail sex, reproductive mode and infection status (Table 1) when testing for geographic associations with microbiota by only conducting analyses on female uninfected asexual snails, allowing us to directly compare native and invasive snails without the confounding factors of sex, reproductive mode and infection (comparisons of these factors are described below). For alpha diversity analyses, we rarefied samples to 10,000 ASVs per sample and discarded two samples that had fewer reads than this threshold. To test covariate effects on microbiota alpha diversity, we used Welch's two‐sample *t* tests and adjusted *p*‐values (“adj‐*p*”) with a Bonferroni correction for multiple tests. We conducted beta diversity analyses on all ASVs after removing singletons. We normalized ASV counts by adding one and then log_e_‐transforming ASV counts (Callahan et al., 2016). To evaluate beta diversity, we performed PCoA on the distance matrices built on the unweighted and weighted UniFrac scores of each sample (Lozupone & Knight, 2005).

We used Analysis of Similarity (ANOSIM; 999 permutations, R‐statistics (*R* ^2^) and exact *p*‐values reported in Results) to evaluate effects of geographic location (sample site) on microbiota beta diversity. Because all European snails were female uninfected asexual snails and many New Zealand snails were sexual, infected and/or male, we only performed the ANOSIM comparing Europe and New Zealand on female uninfected asexual snails.

To analyse population specificity, we focused on the core microbiome of snails within Europe or New Zealand, defined as taxa found in at least 90% of samples within Europe or New Zealand. We used a *t* test to compare the proportion of reads that mapped as core microbiome taxa between the combined Europe and combined New Zealand sample groupings.

We performed a Permutational Multivariate Analysis of Variance Using Distance Matrices analysis (ADONIS) to test the effects of reproductive mode, sex and infection status on microbiota beta diversity. The ADONIS analysis was limited to the Lake Alexandrina (New Zealand) sample site as it was the only site for which we were able to obtain all conditions (sexual/asexual, male/female, infected/uninfected). ADONIS tests were conducted with 999 permutations. We calculated the sample distance from European and New Zealand beta diversity centroids using the vegan's betadisper function. We tested the hypothesis that the average distance to group centroids was different between European and New Zealand snails, where groups were Europe and New Zealand, by performing a Permutation test for homogeneity of multivariate dispersion with 999 permutations.

We employed the Analysis of Composition of Microbes (ANCOM) algorithm to conduct a differential count analysis (Mandal et al., 2015). These analyses were limited to the Lake Alexandrina (New Zealand) sample site for the reasons noted above. We tested the effects of sex, reproductive mode and infection status on the microbiome in uninfected male versus uninfected female sexual snails, uninfected female sexual versus uninfected female asexual snails and asexual female trematode‐infected versus asexual female uninfected snails, respectively. We used machine learning to model microbiome classification by geography using female uninfected asexual snails and ASVs agglomerated phylogenetically using default settings (phyloseq tax_glom; h = 0.2). Machine Learning was trained on a random forest model using the caret package (v6.0–81) with a test split of 80:20 and fit a random forest classifier over the tuning parameter of snail origin (New Zealand or Europe). Please see the Electronic Supplemental Methods for more details on bioinformatic and statistical analyses.

## 3 RESULTS

**Figure 1:**
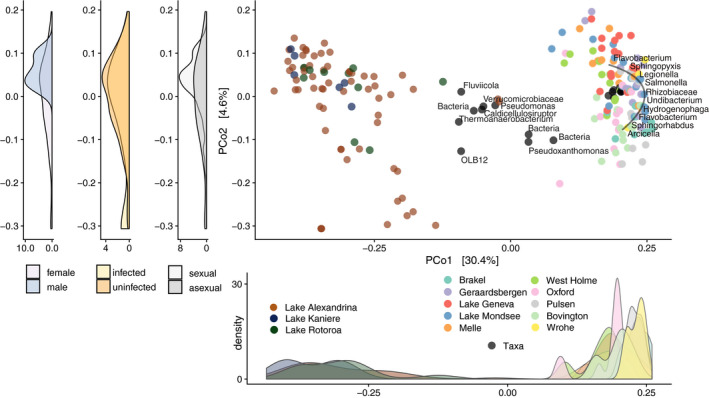
Snail microbiota ecosystem clustering. Biplot PCoA on unweighted UniFrac dissimilarity distances among snail microbiota profiles across European and New Zealand sample sites. Each point represents a single snail and points are colour‐coded based on whether a point is a microbial taxon (grey filled with outline colour indicating sample site) or snail (colour filled by sample site) and indicates collection site. Taxa represented on the plot are the top 20‐ranked differentials in predicting whether a snail was from New Zealand or Europe and are annotated at the deepest available taxonomic level. Density plot below PCo1 shows snail sample density across PCo1 labelled by sample site. Density plots to the left of PCo2 show snail sample density across PCo2 labelled by sex (male or female), infection status (infected or uninfected) and reproductive mode (sexual or asexual)

Variation in *P. antipodarum* microbiota was in large part driven by whether a snail was collected from Europe or New Zealand (Figure 1; ANOSIM; *R* = .97*; p* < .01; Permutations: 999; ADONIS; *R* ^2^ = .33, *p* < .01; weighted UniFrac dissimilarity distances are presented in Figure S2). To validate this analysis, we performed a Mantel test on coordinate and microbiota distance matrices and found geographic distance was significantly associated with microbiome distance (Mantel test; Observation = 0.13; *p* < .01). Within the two European and New Zealand regions, we did not find significant differences in microbial variation among sample sites. We thus attribute the geographic distance‐to‐microbiome distance correlation to major differences between microbiomes from the most geographically distant regions of Europe and New Zealand, rather than differences among sites within the two larger regions. We also observed that the average distance from beta diversity centroids was higher in New Zealand snails (mean distance: 0.437) than European snails (mean distance: 0.353). This result suggests that beta diversity among New Zealand snails is higher than that among European snails (Permutation test for homogeneity of multivariate dispersions; permutations: 999; *p* < .01). To confirm these results were not due to uneven sampling (10 European sample sites versus 3 New Zealand samples sites), we permuted PCoA on the distance matrices built on the unweighted UniFrac on all combinations of three randomly selected Europe sites and the three New Zealand sites (Figure S3). A bar chart of microbial taxa at the family level for each sample site is presented (Figure S4).

To identify taxa that significantly predicted ecosystem clustering, where ecosystems are defined by the two regions of Europe and New Zealand, we used machine learning classification via a random forest model to classify snails based on microbiome composition. The model classified samples based on relative microbial abundance into European or New Zealand ecosystems with 99.6% accuracy. We corroborated these results using a reference frames approach (Morton et al., 2019) and identified taxa associated with snails from the two regions. Eight phyla were exclusively associated with European snails and none with New Zealand snails (Table 2; Figure 1). However, there were more Firmicutes taxa associated with snails from New Zealand (9 taxa) than Europe (6 taxa) (Table 2; Table S1).

**Table 2:** Europe and New Zealand microbiome phyla predictors

Phylum	Europe	New Zealand
Acidobacteria	3	0
Actinobacteria	4	0
Armatimonadetes	2	0
Bacteroidetes	31	13
Chlamydiae	0	0
Chloroflexi	0	0
Cyanobacteria	4	3
Deinococcus‐Thermus	1	0
Epsilonbacteraeota	1	0
Firmicutes	6	9
Gemmatimonadetes	0	0
Lentisphaerae	0	0
Nitrospirae	1	1
Planctomycetes	2	0
Proteobacteria	65	32
Spirochaetes	1	0
Tenericutes	1	0
Verrucomicrobia	1	2

Note

We used a multinomial regression to predict whether snails were from Europe or New Zealand samples sites.

Represented here is a summary of counts of phyla that were associated with snails from EU or NZ.

**Figure 2:**
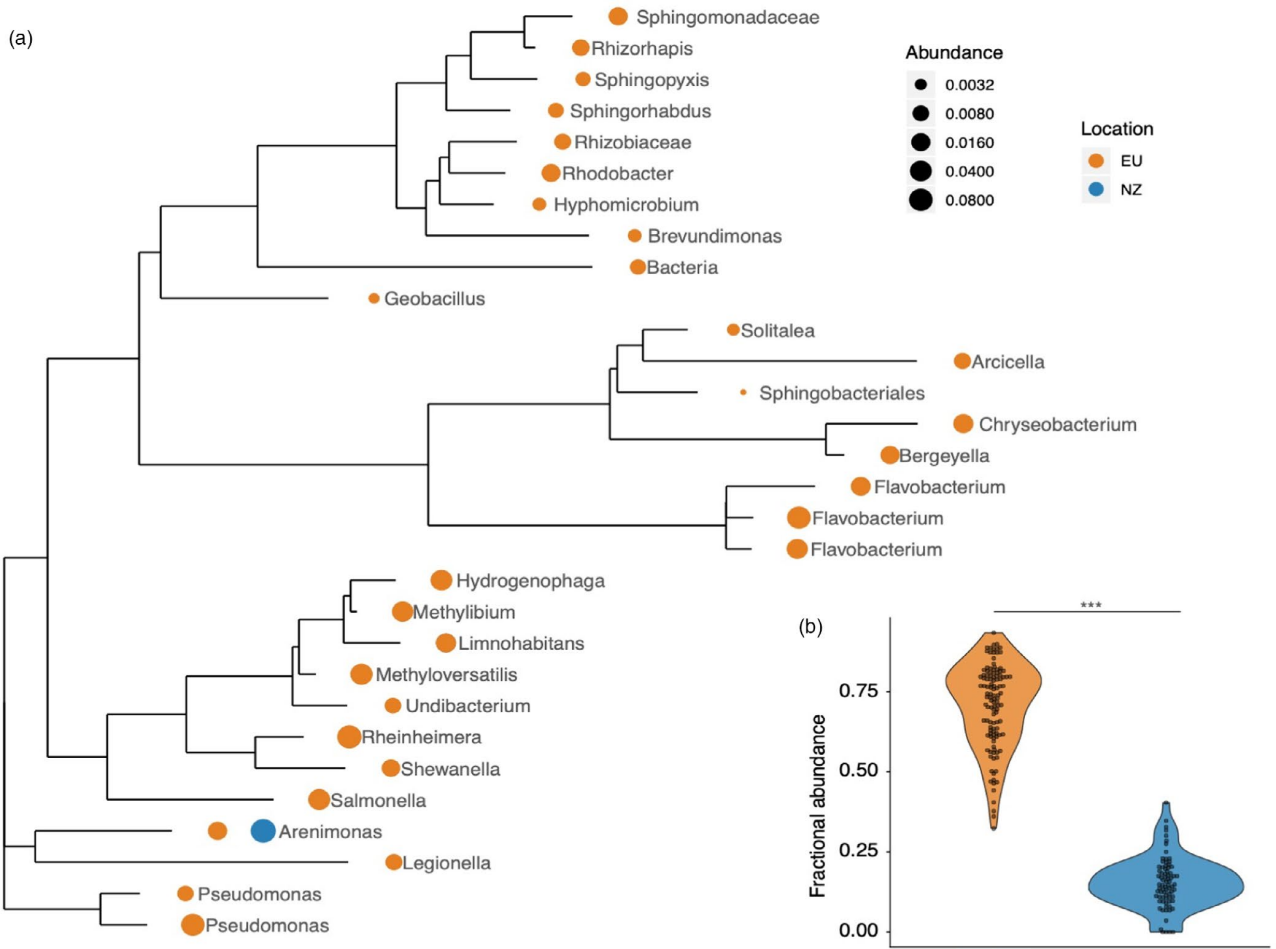
Core microbiota of European and New Zealand snails. (a) 16S rRNA phylogeny of core microbiota (>90% samples) of European (EU) and New Zealand (NZ) snails. Fractional abundance averaged across samples within the EU and NZ regions is represented by dot size. (b) Violin plot summarizing the proportion of amplicon reads mapped to core. Significance is denoted as *** = *p* < .001 (*t* test)

To test the degree to which microbiota exhibit host population‐specific distributions, we calculated the core microbiome of snails from Europe and New Zealand, defined at the tip agglomerated level. European snails had a core microbiome (found in >=90% of samples) of 30 taxa while snails from New Zealand had one core taxon (Figure 2a), *Arenimonas*, also in the European snail sore microbiome. Our results thus departed from the prediction of higher core microbiome specificity for in the New Zealand region. We found that the core microbiome of European snails constituted, on average, 13.8% of their microbiome. By contrast, the core microbiome of New Zealand snails constituted 70.1% of their microbiome, a marked and statistically significant difference between native and invasive snails (Figure 2b; *t* test*; p* < .01). Proteobacteria were the main constituent of the core microbiota in both snail populations, making up 100% (1/1) of the New Zealand snail core and 68.9% of the taxa in the European snail core (20/29).

**Figure 3:**
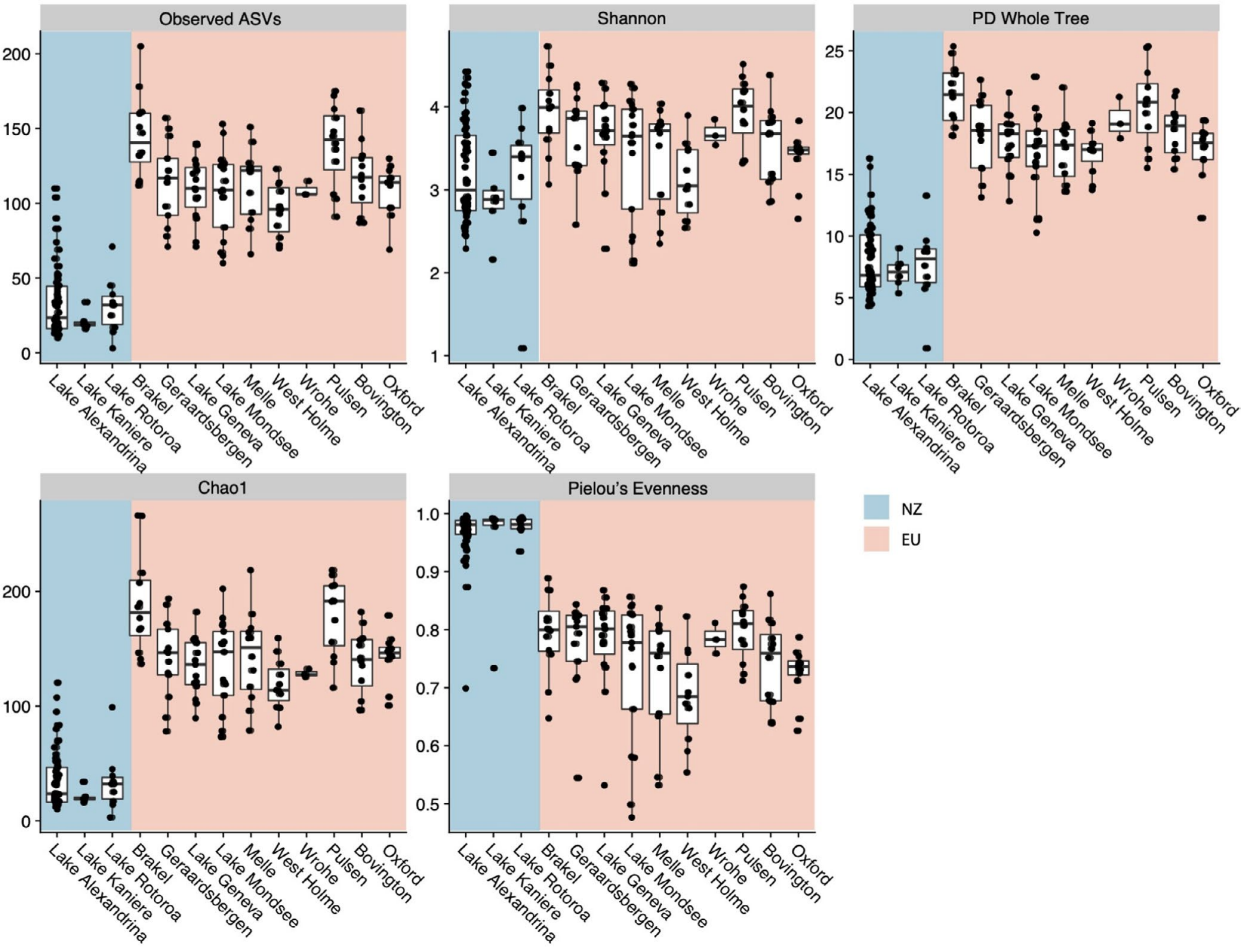
Alpha diversity measurements across sample sites. Figures are faceted by diversity metric. X‐axis shows sampling site. Y‐axis shows value of diversity metric. NZ, New Zealand samples and EU, European samples

We next evaluated whether there were differences in species richness between European and New Zealand snail microbiota. We found that European snail microbiota (mean: 116 ASVs, se: 2.51) had on average 3.52x more ASVs than those in New Zealand (mean: 32.9 ASVs, se: 2.56) (Figure 3; *t* test; adj*‐p* < .01; *t* = −23.1). There was significantly higher Chao1 and PD Whole Tree diversity among European snail microbiota, suggesting higher richness of rare species at relatively low abundance and higher phylogenetic diversity (Figure 3; *t* test; adj*‐p‐*values < 0.01; Chao1 *t* = −25.2; PD *t* = −24.8). Shannon diversity was also significantly higher in European snail microbiota (Figure 3; *t* test; adj*‐p* < .01; *t* = −4.52). Our prediction that New Zealand snail microbiota may have higher Pielou's evenness than the European snails was upheld (Figure 3; *t* test; adj*‐p* < .01; *t* = 22.4). To confirm that alpha diversity differences between snail microbiota in Europe and New Zealand were not due to uneven sampling, we permuted beta diversity tests on all combinations of three, randomly selected Europe sites versus the three New Zealand sites (Figure S3). Among the 600 combination tests, 98.3% remained significant (Table S2; *t* test; adj*‐p* < .05). The nonsignificant findings were for Shannon diversity comparisons between European sites including West Holme and Melle compared with New Zealand sites. There were thus more species, more rare species and higher phylogenetic richness in European snail microbiota, but higher species evenness in New Zealand snail microbiota.

Our comparisons of snails from Lake Alexandrina show that sex (*R* ^2^ = .147; adj‐*p* = .001), reproductive mode (*R* ^2^ = .0467; adj‐*p* = .001) and infection status (*R* ^2^ = .0330; adj‐*p* = .005) all are associated with microbiota composition (Table 3; ADONIS; Permutations: 999). We controlled for these covariate effects and highlight taxa that significantly differed in abundance by sex and reproductive mode (Figure S5; ANCOM; adj‐*p* < .05). No taxa significantly differed in abundance based on infection status. We identified ten ASVs (all Xanthomonadaecae) that were significantly higher in abundance in asexual versus sexual snails (Figure S5). We observed 50 ASVs (*Niabella* (*n* = 16), *Bacillus* (*n* = 15) and OM60 (*n* = 19)) that were significantly more abundant in male versus female snails. There was significantly higher ASV richness in uninfected compared with infected snails (*t* test; adj*‐p* < .01; Figure S6). There were no significant differences in observed ASV richness when comparing reproductive mode or sex (Figure S6).

**Table 3:** ADONIS testing effects of sex, ploidy and infection status on microbiome UniFrac distances

Condition	*df*	SS	MS	*F* Model	*R* ^2^	*p*
Sex	1	1.2899	1.28991	14.5946	.14665	.001
Reproductive mode	1	0.4105	0.41049	4.6444	.04667	.001
Infection	1	0.29	0.29	3.2813	.03297	.005
Residuals	77	6.8055	0.08838	0.77371		
Total	80	8.7959	1			

Note

Results from Analysis of Variance Using Distance Matrices (ADONIS) testing of the effects of sex, ploidy and infection status on unweighted microbiome UniFrac distances between snails.

All tests were performed on snails from Lake Alexandrina with 999 permutations.

## 4 DISCUSSION

As organisms invade new habitats, resident microbes can be lost (Minard et al., 2015; Zepeda‐Paulo et al., 2018). Microbes might alternatively be gained if new associations are readily formed, possibly with less host control (Foster et al., 2017). We predicted that invasion by a host organism would be reflected in changes to the composition and/or diversity of its microbiota. We found that native New Zealand and invasive European snails had distinct microbiotas. While invasive snails carried many core taxa from New Zealand, they also harboured more core members overall and higher phylogenetic diversity. These results suggest that invaders can form many new microbial associations, which may play a role in the success of this worldwide invasive snail. The results also suggest that microbes inhabiting invasive snails may be opportunistic and less specialized. This pattern may be a consequence of the comparatively short time for host‐microbiome co‐adaptation, relative to the potential for longer coevolutionary relationships between New Zealand snails and microbes in the native range. In new environments, host‐control mechanisms may not have yet evolved to keep microbial diversity in check (Foster et al., 2017).

The single core microbial taxon from native New Zealand snails (*Arenimonas*) was found in European invasive snails. There could thus be selection to maintain certain symbiont associations in both ranges, which may be important to host biology. Because we were unable to obtain environmental samples due to insufficient amounts of DNA in water samples, and it is unclear whether some core microbes are inherited, we cannot say whether core microbes found in European and New Zealand snail populations were present in both environments, or carried over from the latter. Nevertheless, European samples had 30 times more core taxa overall, and their most predictive taxa were absent or relatively infrequent in native snails. In other systems, it has been shown that invaders have lower microbial diversity and/or a subset of microbes relative to that present in native populations (Minard et al., 2015; Zepeda‐Paulo et al., 2018). Invasive *P. antipodarum* populations feature low genotypic diversity relative to the native range (Donne et al., 2020; Dybdahl & Drown, 2011; Verhaegen et al., 2018) and mitochondrial haplotypes of invaders represent genetic subsets of native mitochondrial haplotype diversity (Städler et al., 2005). However, this genetic subsampling in invasive *P. antipodarum* populations is not reflected in their microbiota diversity.

Across both ranges, Proteobacteria dominate among the core microbes. Proteobacteria and the majority of the other core microbial taxa observed here are consistent with previous work performed in this species by Takacs‐Vesbach et al. (2016). The predominance of Proteobacteria among the core microbiome, combined with the reduced alpha diversity in New Zealand, suggests a possible shared coevolutionary history between snails and their resident microbes. Coevolution and evolved host‐control mechanisms could drive the loss of nonessential partners and ultimately streamline the microbiome down to specialized taxa (Foster et al., 2017). A similar phenomenon occurs in a number of insect systems, whereby the host harbours few core microbes that are key players in organismal function (Chandler et al., 2011; Raymann & Moran, 2018). Conversely, other insect taxa are host to relatively diverse core microbial populations (Tinker & Ottesen, 2016).

Coevolution between *P. antipodarum* and *A. winterbourni* is well documented (Dybdahl & Lively, 1996; King et al., 2009; Lively, 1987). Reciprocal antagonistic selection between *P. antipodarum* and *A. winterbourni* is expected to be strong because infected snails are completely sterilized (Winterbourn, 1973), and parasites die if they encounter resistant individuals (King et al., 2011). In other systems, the potential for beneficial microbes to be involved in host defence and even host‐parasite coevolution has been demonstrated (Dillon et al., 2005; Ford & King, 2016; Vorburger & Perlman, 2018). However, the extent to which coevolution operates in diverse communities that feature the microbiome. Our results do not suggest that the microbiota is a dominant player in this host‐parasite coevolutionary relationship: some taxa differed only marginally, but not statistically significantly, in relative abundance between infected and uninfected snails, although we did observe significantly higher ASV richness in uninfected snails compared with infected snails.

Because asexual *P. antipodarum* are more successful invaders than sexuals, whether the microbiome could influence the success of asexual invaders is of interest. Here, we found that reproductive mode was associated with microbiome composition and ten ASVs that were significantly more abundant in asexuals than sexuals. Takacs‐Vesbach et al. (2016) observed significant differences in microbiome composition between sexuals and asexuals and among lake populations of *P. antipodarum*. Because the mechanism(s) leading to transitions from diploid sexuality to polyploid asexuality in *P. antipodarum* are still unclear, future work could focus on the possible links between microbes and reproductive mode and/or polyploidization. For example, *Wolbachia*‐mediated transitions to asexuality are well characterized in arthropods (Boivin et al., 2014). In regard to potential effects of ploidy level, Takacs‐Vesbach et al. (2016) did not observe significant differences in microbiome composition between asexual triploids and tetraploids, and we were unable to obtain sufficient sampling of tetraploids to perform ploidy level comparisons. Furthermore, besides a handful of studies (Cavé‐Radet et al., 2019; Forrester & Ashman, 2017), little is known about links between host ploidy level and microbiome composition, especially in animals.

While it is established that resident microbes have important effects on host fitness, the relationship between microbes and invaders has received little attention. Here, we find that the process of invasion is associated with some carryover of a core microbiota, but mostly involves the formation of numerous new microbial relationships, indicating that successful invaders, like *P. antipodarum*, might take advantage of available microbes. Despite some impact of biological trait variation on the microbiota in the native range, there is a consistent and strong pattern highlighting that long‐term host–microbiota interactions, and possibly coevolution (or host evolution alone (Foster et al., 2017)), could streamline the microbiota. Future research will need to establish the fitness impacts of those new microbes on invaders, and whether the invasive snails have adapted to accommodate them. Notwithstanding this possibility, the potential for invasion to alter the host microbiome is clear. Endeavours to control invasive species could focus on these new associations being formed with microbes and investigate their contribution to invader success. The “make or break” of an invasion may hinge upon the ability of an introduced species to take advantage of the new microbes it encounters.

## CONFLICT OF INTERESTS

None declared.

## Supporting information

Figure S1‐S6

Table S1

Table S2

Appendix S1

## Data Availability

Raw sequencing data are publicly available on the European Nucleotide Archive under project accession PRJEB40881, and sample accessions ERS5219283‐ERS5219479 (SAMEA7461104‐SAMEA7461300).
