# Supplementary material for: Invasive freshwater snails form novel microbial relationships
Source: Evol Appl. 2020 Nov 20;14(3):770–80. doi: 10.1111/eva.13158 (PMC7980272; doi:10.1111/eva.13158)
Supplement: Supplementary file 1 — Figure S1‐S6 [file EVA-14-770-s003.pdf]

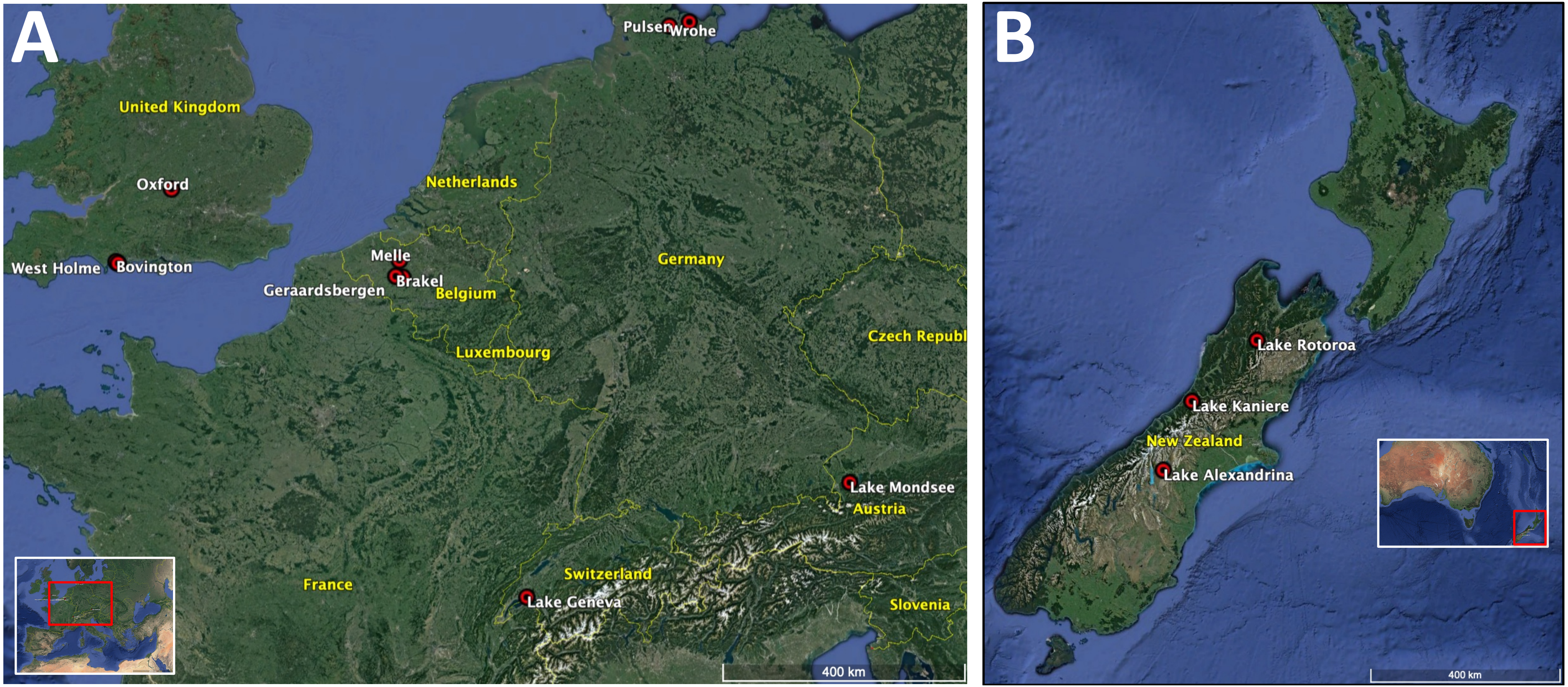

Fig. S1. Map of sampling sites. (A) Locations of our ten European sampling sites. (B) Locations of our three New Zealand sampling sites. Country names and boundaries are in yellow. Sampling sites are represented by red dots and the location names are in white. Zoomed out inlayed maps show the broader regions with the sampled regions highlighted in the red boxes. Maps were made using Google Earth Pro v. 7.3.2.5776.

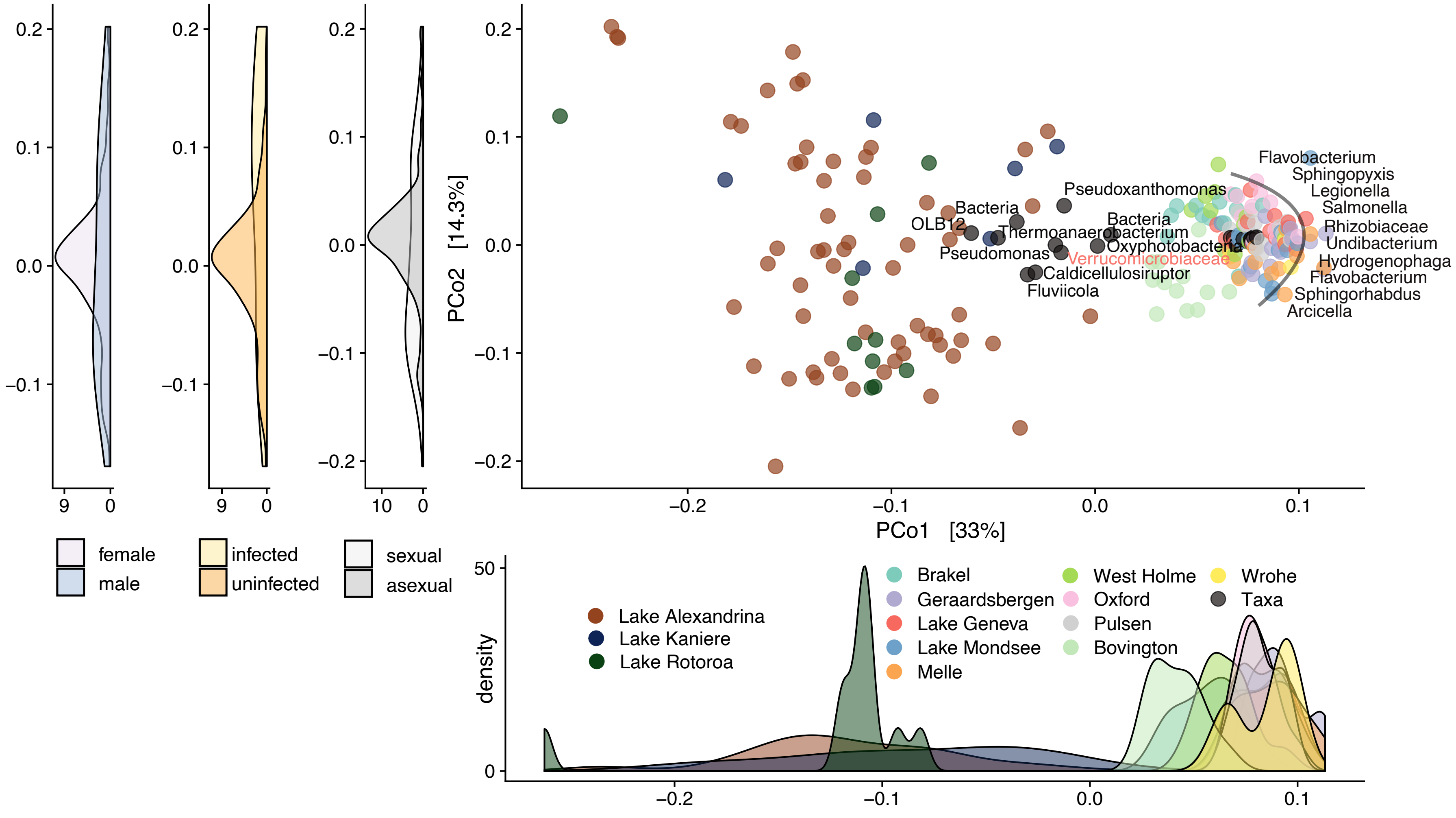

Fig. S2. Snail microbiota ecosystem clustering. Biplot PCoA on weighted UniFrac dissimilarity distances between snail microbiota profiles across European and New Zealand sampling sites. Each point represents a single snail, and points are colour-coded by whether a point is a microbial taxon or snail and indicates snail collection site. Taxa represented on the plot are the top 20-ranked differentials in predicting whether a snail is from New Zealand or Europe and are annotated at the deepest available taxonomic level. Density plot below PCo1 shows snail sample density across PCo1 labelled by sampling site. Density plots to the left of PCo2 show snail sample density across PCo2 labelled by sex (male or female), infection status (infected or uninfected), and reproductive mode (sexual or asexual).

Fig. S3. Combination PCoAs on unweighted UniFrac dissimilarity distances between snail microbiota profiles across European and New Zealand sample sites. Each point represents a single snail, and points are colour-coded by collection site of snail and shaped by whether a snail is from Europe or New Zealand. Include all combinations of snails from three European sites compared to snails from all three New Zealand sites.

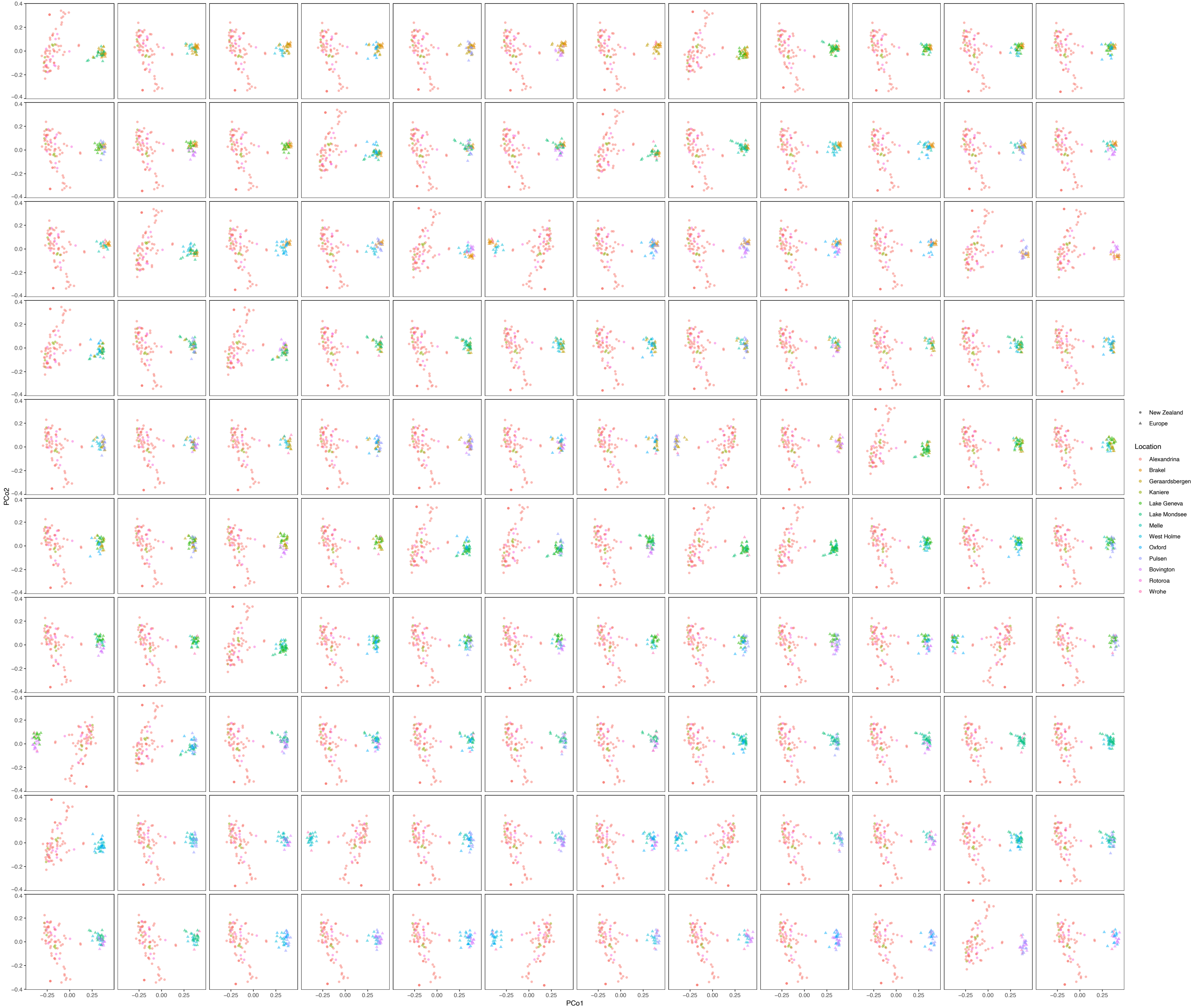

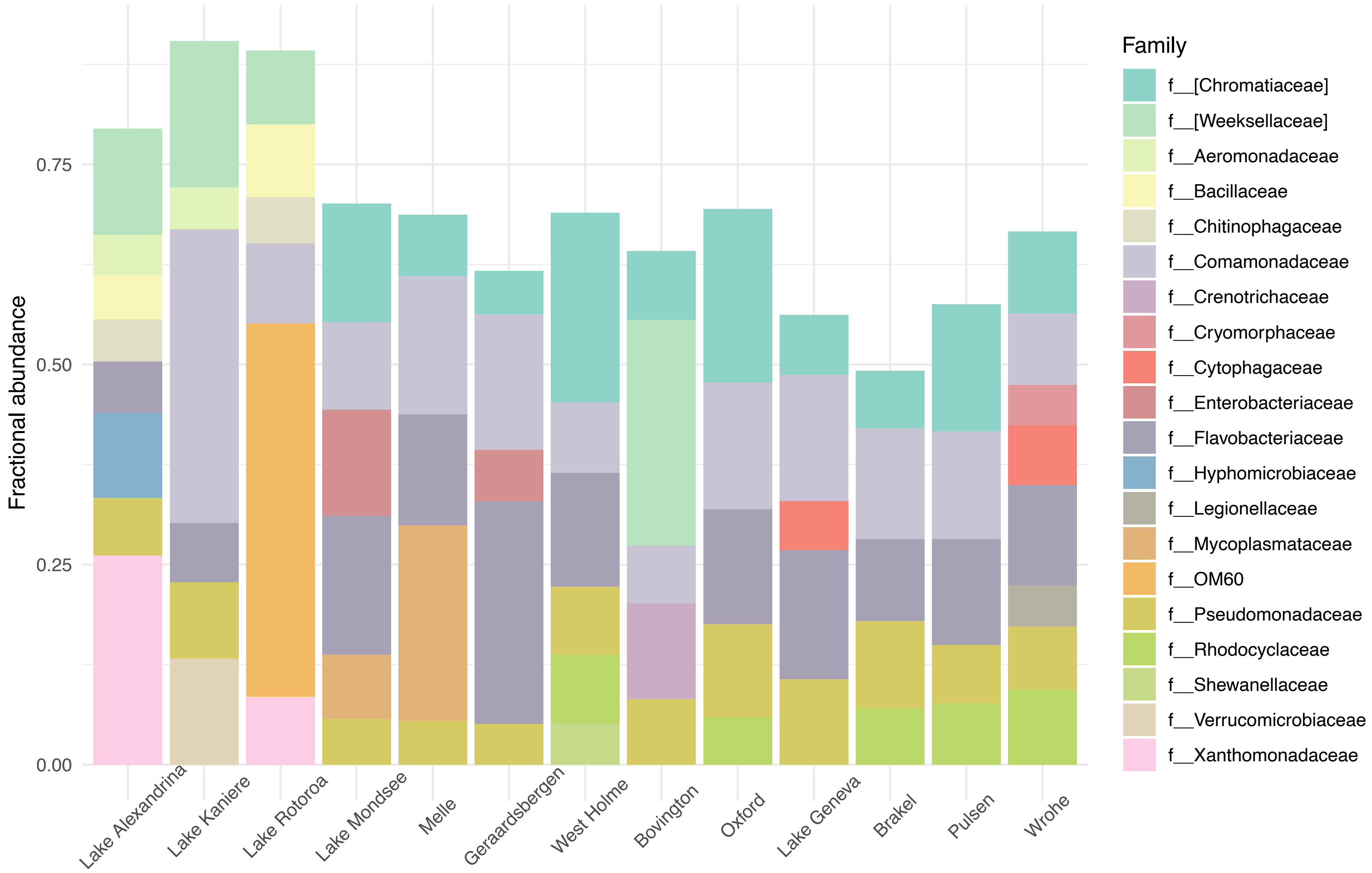

Fig. S4. Stacked barplot annotated at the family level. Mean fractional abundance of microbial families are plotted per sampling site. Only showing families with at least 5% abundance in at least one location.

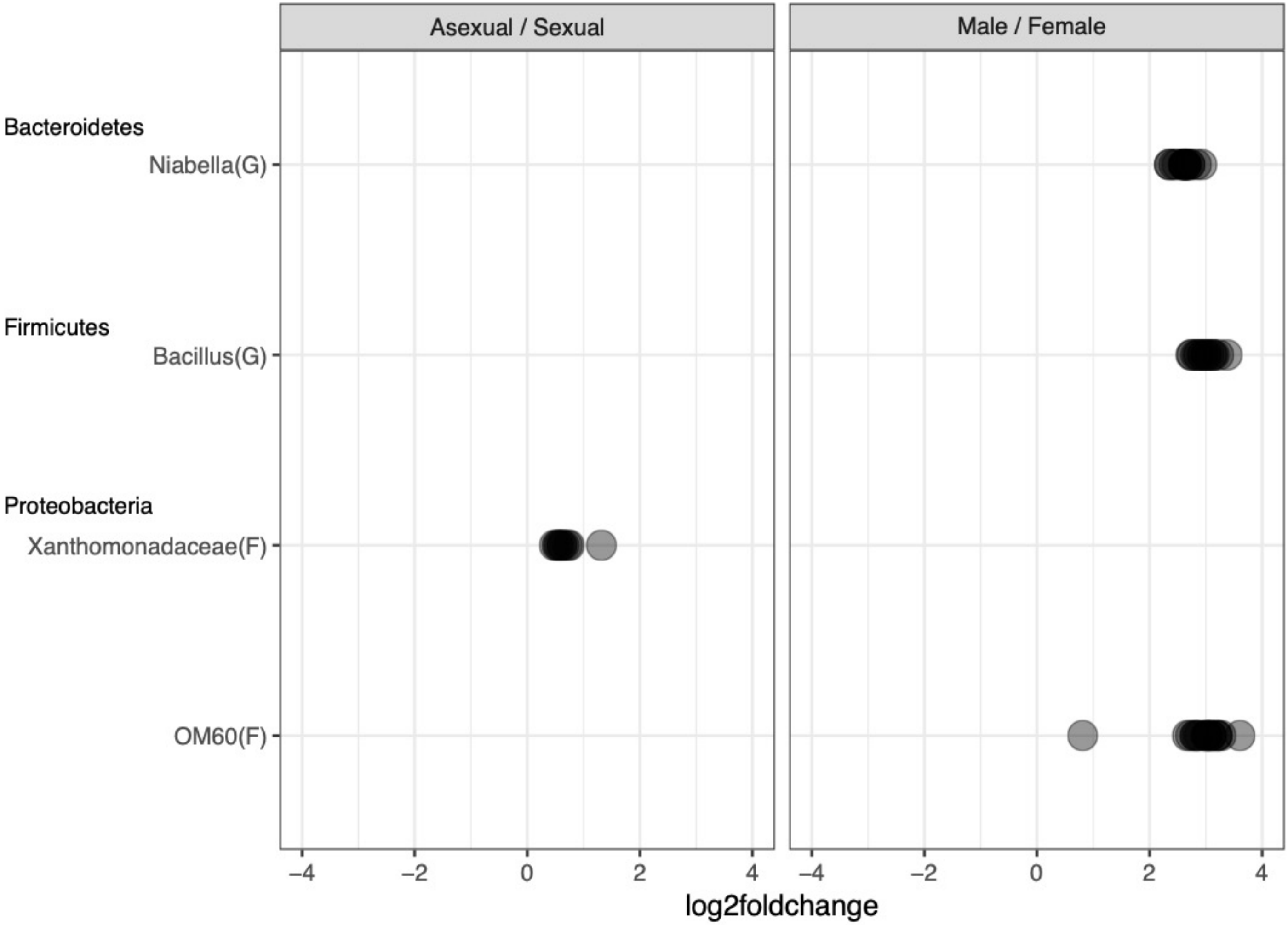

Fig. S5. ASVs that significantly differed in abundance in snail microbiota based on reproductive mode or sex. Points represent significantly differentially abundant taxa and are plotted by log2fold change in abundance (ANCOM; adj-p < 0.01). Comparisons are asexual/sexual and male/female. There were no significantly differentially abundant taxa between infected and uninfected snails. Figure is labelled by phyla and deepest available taxonomy, where G = genus and F = family. Snails are only from Lake Alexandrina, NZ.

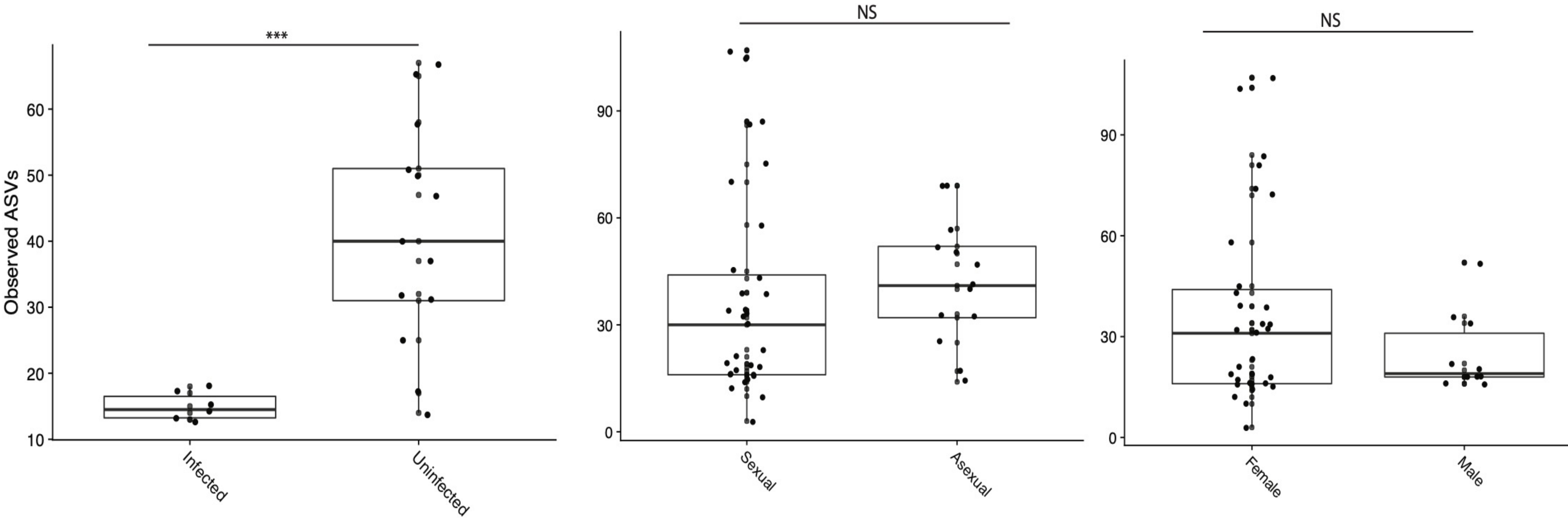

Fig. S6. Observed ASVs faceted by infection status, reproductive mode, and sex. Each point represents a snail sample. Comparing observed ASVs, as a measure of species richness, across snail metadata variables of infection status, reproductive mode, and sex. Data from Lake Alexandrina sails, the only collection site where all metadata variables were tested (T-test; *** = adj-p < 0.01).
